# The association between waist-to-hip ratio (WHR) with diabetes in the PERSIAN Guilan cohort study population

**DOI:** 10.1186/s12902-024-01641-1

**Published:** 2024-07-15

**Authors:** Masoome Aghaei, Farahnaz Joukar, Soheil Hasanipour, Zahra Abbasi Ranjbar, Mohammadreza Naghipour, Fariborz Mansour-Ghanaei

**Affiliations:** Gastrointestinal and Liver Diseases Research Center, Guilan University of Medical Sciences, Rasht, Iran

**Keywords:** Diabetes, WHR, BMI, Type 2 diabetes mellitus

## Abstract

**Background:**

Waist circumference (WC), or waist-to-hip ratio (WHR), potentially offers a more accurate reflection of intra-abdominal fat accumulation and could serve as a superior predictor of type 2 diabetes mellitus (T2DM) risk compared to BMI. The current study investigated the relationship between WHR and its influencing factors among diabetes patients enrolled in the Prospective Epidemiological Research Studies in Iran (PERSIAN) Guilan Cohort study (PGCS).

**Method:**

In this cross-sectional study of 10,520 participants, 2,531 had T2DM. Waist and hip circumference, body mass index (BMI), underlying diseases, and demographical data of participants were recorded. Also, fasting blood sugar (FBS), low-density lipoprotein (LDL) cholesterol, high-density lipoprotein (HDL) cholesterol, and triglycerides (TG) were assessed. All data was analyzed using SPSS version 16; the significant level was < 0.05.

**Results:**

The mean age of participants was 51.52 ± 8.90 years, and 39.9% had a BMI between 25 and 30 kg/m^2^. The prevalence of diabetes was 24.1% (*n* = 2531). About 7628 (72.5%) individuals had abnormal WHR, and 2072 (19.7%) were diabetics. Among patients with diabetes, abnormal WHR was significantly associated with age over 50, female gender, higher BMI, and lower LDL (*P* < 0.05).

**Conclusion:**

The study showed a higher prevalence of abnormal WHR in diabetic patients. Abnormal WHR in patients with diabetes was significantly associated with age, gender, and BMI.

## Introduction

Diabetes has emerged as a substantial global public health challenge in recent years, with the worldwide prevalence reaching 10.5% and projected to rise to 12.2% by 2045. The most notable increase in diabetes prevalence has been observed in middle-income countries [1, 2]. Both genetics and epigenetics play a significant role in the onset of diabetes and following clinical and daily life complications [3, 4]. The predictive efficacy of obesity-related variables for diabetes onset may vary across ethnicities, age groups, and genders, highlighting the role of both genetics and epigenetics in vulnerability to diabetes [5, 6].

Obesity poses a critical health threat, significantly increasing the risks of coronary heart disease, stroke, and type 2 diabetes mellitus (T2DM). However, body mass index (BMI) has been commonly used to evaluate weight status, but it inadequately assesses cardiometabolic risks in adults with excessive adiposity [7, 8]. In this regard, studies indicated that BMI, waist circumference (WC), and waist-to-hip ratio (WHR) anthropometric index are comparable in predicting diabetes onset and following complications [9, 10]. Moreover, patients with diabetes usually illustrated abnormalities in their laboratory findings [11, 12]. The WHR associated with fasting blood sugar (FBS) levels, proteomic profile, and lipid profiles reflect its role in assessing metabolic health. Studies have consistently demonstrated that individuals with disrupted anthropometric indicators often exhibit elevated FBS levels and, on the other side, diabetes associated with dyslipidemia [13, 14].

Moreover, a higher WHR is frequently correlated with adverse lipid profiles characterized by elevated levels of triglycerides (TG) and low-density lipoprotein (LDL) cholesterol and decreased levels of high-density lipoprotein (HDL) cholesterol [15, 16]. These lipid abnormalities contribute to a heightened risk of cardiovascular diseases among individuals with central adiposity, where WHR is a helpful predictor [17, 18]. Due to the importance of demographical and clinical risk factors associated with T2DM and the critical role of anthropometric indicators in the severity of the condition, by considering the limited studies on this issue in north Iran, the current study investigated the prevalence of T2DM and the relationship between WHR and diabetes in the Prospective Epidemiological Research Studies in Iran (PERSIAN) Guilan Cohort study (PGCS) population and the related risk factors.

## Methods

### Study design and participants

This cross-sectional study was derived from the data of the PERSIAN cohort study [19]. The study encompassed 10,520 individuals from the PGCS cohort, among whom 2,531 were diagnosed with diabetes and 7,989 were non-diabetic participants [20]. The demographical and clinical data, including age, gender, educational status, history of smoking, and hypertension were recorded. Anthropometric indicators, including weight (kg), height, hips, and WC, were measured according to the National Health and Nutrition Examination Manual [21]. BMI was reported as low weight (BMI < 18.5 kg/m^2^), average weight (BMI = 18.5–24.99 kg/m^2^), overweight (BMI = 25–29.9 kg/m^2^), and obese (BMI ≥ 30 kg/m^2^). WHR was calculated by dividing WC by hip circumference, and the normal level of WHR was evaluated as 0.9 or less in men and 0.85 or less in women.

The MET was used to assess the intensity of physical activity based on a standardized classification of the energy costs of different physical activities. MET was calculated using questionnaires given to the participants in face-to-face interviews by trained people to measure their activity intensity. The oxygen that was used during rest and immobility was equal to MET, and the participants were divided into four quartiles as sedentary, light, moderate, and high levels of physical activity according to daily activity levels by the number of hours of walking, working, exercise, etc. [19]. Hypertension was defined as a systolic blood pressure ≥ 140 mm Hg or a diastolic blood pressure ≥ 90 mm Hg [20].

The laboratory findings, including FBS, TG, LDL, and HDL, were analyzed via a Biotechnical auto-analyzer (BT 1500, Italy) at the medical laboratory of the PGCS center. Diabetes was definite as FBS ≥ 126 mg/dL and/or a history of diabetes diagnosis or use of anti-diabetic medications. Abnormal lipid profile was defined as follows: Chol ≥ 200 U/L, TG ≥ 150 U/L, LDL ≥ 100 U/L, and HDL ≤ 40 U/L [22].

### Statistical analysis

Data was reported as mean ± standard deviation (SD) and number (percentage). The association between WHR and diabetes was analyzed using logistic regression analysis by odds ratio (OR) with a 95% confidence interval (CI). All data was analyzed using SPSS for Windows, version 16.0 (SPSS Inc., Chicago, IL, USA), and the significance level was set at 0.05.

## Results

The mean age of participants was 51.52 ± 8.90 years. About 53.5% were female, 39.9% had normal BMI and 2531(24.1%) individuals had diabetes. A total number of 7628 (72.5%) people had abnormal WHR, of which 2072 (19.7%) people with diabetes and 5556 (52.8%) non-diabetics had abnormal WHR. Abnormal WHR was prevalent in most diabetic individuals aged over 50 and in most non-diabetic people under 50 (*P* > 0.05). In the diabetics group with abnormal WHR, 73.5% were females, and in non-diabetics, 70.7% of individuals with abnormal WHR were females. Most of the diabetic patients with abnormal WHR had abnormal levels of HDL and TG (71.0% and 72.0%, respectively), while the majority of non-diabetics had abnormal HDL (82.4%) (Table 1).

**Table 1:** Comparison of demographical and clinical data of diabetic and non-diabetic individuals with normal and abnormal WHR

		Diabetic (%)		*P*-value	Non-Diabetic (%)		*P*-value
Demographical data		Normal WHR	Abnormal WHR		Normal WHR	Abnormal WHR	
**Age**	< 50 years	190 (41.3)	659 (31.7)	< 0.001	1442 (59.2)	2945 (53)	0.001
	> 50years	269 (58.6)	1416 (68.2)		991 (40.7)	2611 (46.9)	
**Gender**	Male	440 (95.8)	549 (26.4)	< 0.001	2272 (93.3)	1626 (29.2)	0.001
	Female	19 (4.1)	1523 (73.5)		161 (6.6)	3930 (70.7)	
**Education**	Illiterate	55 (11.9)	581 (28.0)	< 0.001	211 (8.6)	891 (16.03)	0.001
	Elementary	147 (32.0)	661 (31.9)		649 (26.6)	1855 (33.3)	
	Diploma	225 (49.0)	749 (36.1)		1334 (54.8)	2524 (45.4)	
	University degree	32 (6.9)	81 (3.9)		239 (9.8)	286 (5.1)	
**MET**	Sedentary (Q1)	101 (22.0)	701 (33.8)	< 0.001	444 (18.2)	1380 (24.8)	< 0.001
	Light (Q2)	106 (23.1)	572 (27.6)		435 (17.9)	1519 (27.3)	
	Moderate (Q3)	106 (23.1)	491 (23.7)		572 (23.5)	1461 (26.3)	
	High (Q4)	146 (31.8)	308 (14.9)		982 (40.4)	1196 (21.5)	
**Smoking**	No	219 (47.7)	1764 (85.1)	< 0.001	1236 (50.8)	4717 (84.7)	0.001
	Yes	240 (52.2)	308 (14.8)		1197 (49.1)	839 (15.1)	
**BMI**	< 18.5	20 (4.3)	2 (0.1)	< 0.001	103 (4.2)	16 (0.2)	0.001
	18.5–25	248 (54.0)	298 (14.3)		1351 (55.5)	849 (15.2)	
	25–30	151 (32.8)	870 (41.9)		846 (34.70	2331 (41.9)	
	30<	40 (8.7)	902 (43.5)		133 (5.4)	2360 (42.4)	
**Hypertension**	No	215 (46.8)	721 (34.7)	< 0.001	1681 (69.1)	3360 (60.4)	0.001
	Yes	244 (53.1)	1351 (65.2)		752 (30.9)	2196 (39.5)	
**LDL**	Normal	884 (33.3)	1770 (66.7)	< 0.001	181 (16.7)	906 (83.3)	0.090
	Abnormal	1549 (29.0)	3786 (71.0)		278 (19.3)	1166 (80.7)	
**HDL**	Normal	1901 (29.8)	4486 (70.2)	0.004	356 (18.3)	1589 (81.7)	0.700
	Abnormal	532 (33.2)	1070 (66.8)		103 (17.6)	483 (82.4)	
**TG**	Normal	1522 (32.1)	3216 (67.9)	< 0.001	224 (18.0)	1020 (82.0)	0.400
	Abnormal	911 (28.0)	2340 (72.0)		235 (18.3)	1052 (81.7)	

The MET was used to assess the intensity of physical activity based on a standardized classification of the energy costs of different physical activities. It was divided into four quartiles: sedentary, light, moderate, and high physical activity levels

Body mass index (BMI), fasting blood sugar (FBS), triglycerides (TG), cholesterol (Chol), low-density lipoprotein (LDL), and high-density lipoprotein (HDL). Diabetes was definite as FBS ≥ 126 mg/dL and/or a history of diabetes diagnosis or use of anti-diabetic medications. Abnormal lipid profile was defined as follows: TG ≥ 150 U/L, LDL ≥ 100 U/L, and HDL ≤ 40 U/L

Hypertension was defined as a systolic blood pressure ≥ 140 mm Hg or a diastolic blood pressure ≥ 90 mm Hg

According to the logistic regression analysis, all participants over 50 had significantly higher abnormal WHRs than those under 50, *P* < 0.001. While females had abnormal WHR than males in both diabetic and non-diabetic groups, it was higher in the diabetic group (OR = 51.26, 95% CI: 30.11–87.30, vs. OR = 29.88, 95% CI: 24.22–36.85, *P* < 0.001), demonstrating the effect of gender on WHR. The chance of abnormal WHR increased with higher BMI levels in both groups (*P* < 0.05). Abnormal WHR in diabetic patients was significantly associated with lower LDL levels, whereas non-diabetic patients had higher LDL levels (*P* < 0.05). In none of the diabetic and non-diabetic groups, there was no statistically significant correlation with smoking, hypertension, HDL, and TG with abnormal WHR (*P* > 0.05) (Table 2).

**Table 2:** Logistic regression analysis for the Association between demographic and clinical factors among diabetic and non-diabetic individuals with normal and abnormal WHR

Variables		Diabetic				Non diabetic			
		OR (95% CI)				OR (95% CI)			
		Unadjusted	*P*-value	Adjusted	*P*-value	Unadjusted	*P*-value	Adjusted	*P*-value
**Age**	< 50 years	Ref	< 0.001	Ref	< 0.001	Ref	< 0.001	Ref	< 0.001
	> 50 years	1.52 (1.23–1.87)		2.01 (1.48–2.70)		1.29 (1.17–1.42)		2.02 (1.75–2.34)	
**Gender**	Male	Ref	< 0.001	Ref	< 0.001	Ref	< 0.001	Ref	< 0.001
	Female	64.24 (40.1-102-73)		51.26 (30.11–87.30)		34.1 (28.77–40.42)		29.88 (24.22–36.85)	
**Education**	Illiterate	4.17 (2.54–6.83)	< 0.001	1.09 (0.57–2.07)	0.785	3.52 (2.80–4.43)	< 0.001	2.84 (2.05–3.92)	< 0.001
	Elementary	1.77 (1.13–2.77)	0.012	0.81 (0.45–1.40)	0.435	2.38 (1.96–2.89)	< 0.001	1.90 (1.45–2.48)	< 0.001
	Diploma	1.31 (0.85–2.03)	0.218	0.74 (0.44–1.26)	0.279	1.58 (1.31–1.90)	< 0.001	1.45 (1.13–1.85)	0.003
	University degree	Ref	-	Ref	-	Ref	-	Ref	-
**MET**	Sedentary (Q1)	3.29 (2.47–4.38)	< 0.001	1.45 (0.98–2.15)	0.058	2.55 (2.22–2.92)	< 0.001	1.38 (1.14–1.68)	0.001
	Light (Q2)	2.55 (1.92–3.40)	< 0.001	0.95 (0.63–1.42)	0.811	2.86 (2.50–3.28)	< 0.001	1.24 (1.02–1.51)	0.027
	Moderate (Q3)	2.19 (1.64–2.92)	< 0.001	1.29 (0.88–1.90)	0.191	2.09 (1.84–2.38)	< 0.001	1.19 (0.99–1.43)	0.055
	High (Q4)	Ref	-	Ref	-	Ref	-	Ref	-
**Smoking**	No	Ref	< 0.001	Ref	0.566	Ref	< 0.001	Ref	0.341
	Yes	6.27 (5.03–7.81)		1.08 (0.81–1.45)		5.44 (4.88–6.06)		1.07 (0.92–1.25)	
**BMI**	< 18.5	0.004 (0.001–0.020)	< 0.001	0.004 (0.001–0.028)	< 0.001	0.009 (0.005–0.015)	< 0.001	0.006 (0.003–0.012)	< 0.001
	18.5–25	0.053 (0.037–0.076)	< 0.001	0.095 (0.061–0.146)	< 0.001	0.035 (0.029–0.043)	< 0.001	0.047 (0.038–0.060)	< 0.001
	25–30	0.25 (0.17–0.36)	< 0.001	0.47 (0.31–0.72)	< 0.001	0.15 (0.12–0.18)	< 0.001	0.24 (0.19–0.30)	< 0.001
	30<	Ref	-	Ref	-	Ref	-	Ref	-
**Hypertension**	No	Ref	< 0.001	Ref	0.190	Ref	< 0.001	Ref	0.245
	Yes	1.65 (1.34–2.02)		1.20 (0.91–1.59)		1.46 (1.32–1.61)		1.09 (0.94–1.26)	
**LDL**	Normal	Ref	0.093	Ref	0.048	Ref	< 0.001	Ref	0.013
	Abnormal	0.83 (0.68–1.03)		0.75 (0.54–0.99)		1.22 (1.10–1.34)		1.20 (1.04–1.39)	
**HDL**	Normal	Ref	0.689	Ref	0.054	Ref	0.007	Ref	0.095
	Abnormal	1.05 (0.82–1.33)		0.72 (0.52-1.00)		0.85 (0.75–0.95)		0.86 (0.73–1.02)	
**TG**	Normal	Ref	0.869	Ref	0.321	Ref	< 0.001	Ref	0.42
	Abnormal	0.98 (0.80–1.20)		0.86 (0.65–1.14)		1.21 (1.10–1.34)		1.16 (1.01–1.34)	

The MET was used to assess the intensity of physical activity based on a standardized classification of the energy costs of different physical activities. It was divided into four quartiles: sedentary, light, moderate, and high physical activity levels

Body mass index (BMI), fasting blood sugar (FBS), triglycerides (TG), cholesterol (Chol), low-density lipoprotein (LDL), and high-density lipoprotein (HDL). Abnormal lipid profile was defined as follows: TG ≥ 150 U/L, LDL ≥ 100 U/L, and HDL ≤ 40 U/L

Hypertension was defined as a systolic blood pressure ≥ 140 mm Hg or a diastolic blood pressure ≥ 90 mm Hg

## Discussion

Increased body fat contributes to developing metabolic disorders such as T2DM (23). Anthropometric indices measurement is a simple and non-invasive method used for obesity in therapeutic and epidemiological research [23, 24]. Therefore, the present study determined the association between WHR and diabetes. In this study, the prevalence of abnormal WHR in diabetics and non-diabetics was 81.8% and 69.5%, respectively. This finding aligns with other studies that indicate a high prevalence of abnormal obesity in humans [10, 25].

Additionally, most individuals with abnormal WHR were over 50 years old, consistent with the findings of Zhang et al., who confirmed that risk factors associated with diabetes include obesity and age 40 years and above [26]. Hadaegh et al. also found that a BMI of ≥ 30 kg/m^2^ and high WHR were predictors of type 2 diabetes among Iranians under 60 years old [27]. Therefore, age should be considered a confounding factor when using anthropometric measures to predict the risk of T2DM. De Hollander et al. demonstrated that an individual with a higher WC within the healthy weight category has a higher relative risk of mortality compared to an individual with the same WC in the overweight category [28].

Our results showed that females had a higher prevalence of abnormal WHR than males, and this proportion was more significant in individuals with diabetes compared to non-diabetics. Zhang et al. indicated that WC and WHtR are more effective in diagnosing diabetes in women over 40 compared to men [26]. This is likely due to gender differences in visceral fat accumulation and local adipose tissue distribution. Measuring sex hormones may provide further insights into this issue [6, 29, 30]. During menopause, variations in body adipose distribution result in more pronounced abdominal adipose accumulation in postmenopausal women [31]. The association between female visceral adipose tissue and diabetes risk factors is stronger. These findings are consistent with other studies that have identified high WC and WHR as significant risk factors for diabetes in women [32–34].

Education is known to have positive health effects and may prevent obesity through cognitive benefits that lead to healthier lifestyles [35, 36]. We found that abnormalities in WHR scores were more common among individuals with lower levels of education. It can be concluded that people with higher education levels are more concerned about their health because they are more aware and knowledgeable about the complications and side effects of excess fat tissue. As a result, they pay more attention to their diet and lifestyle. However, the association between educational levels and abnormal WHR in diabetics was not statistically significant. This is likely because most diabetic patients receive guidance from nutritionists during treatment, which helps them focus on diet and obesity prevention. Similarly, Chung et al. reported a high prevalence of obesity among less-educated females [37].

We observed higher WHR abnormalities among those with less physical activity. Physical inactivity and obesity are important risk factors for T2DM. A high level of physical activity was associated with a lower risk of diabetes in all BMI categories. Although WHR has been identified as a significant risk factor for physical frailty, especially in the elderly, there is no clear evidence that physical activity could fully offset the negative effects of obesity on diabetes risk [38, 39]. Ali et al. indicated that older age and lower physical activity were significant determinants of general and abdominal obesity [40]. On the contrary, Gutierrez et al. suggested that the WHR and PP are unrelated in this group of military ambulatory hospitals. Contrary to expectations, only women with a higher or altered WHR and older adults aged 60–70 had an appropriate physical performance [41].

The current study showed that the risk of abnormal WHR was lower in diabetic patients with lower BMI. Feller et al. point out that the association between WC and the risk of T2DM was more robust with a low BMI than with a higher BMI [42]. Abdominal obesity is associated with elevated insulin concentrations in younger age groups and impaired glucose control in middle-aged groups [43]. Bala et al. reported that obesity is a risk factor for hemoglobin glycation. Therefore, it should be considered a measure for preventing prediabetes and diabetes [44]. We also found an inverse association between abnormal WHR in diabetics and LDL; no relationship was observed between HDL and TG. Controversially, Choi et al. found a significant positive association between WHR, total cholesterol, and LDL in diabetic men [45]. Sandhu et al. reported positive correlations between WHR, total cholesterol, LDL cholesterol, and TG in the 40–50 age group in diabetic men [46]. In diabetic individuals, the abnormal fat distribution and insulin resistance associated with an elevated WHR may uniquely impact lipid metabolism compared to non-diabetic individuals, leading to the observed differences in LDL cholesterol levels [45]. The current study reported valuable results from a large sample size through a cohort study on the association between diabetes and WHR. At the same time, its cross-sectional nature limited some real-time data.

## Conclusion

The findings found that many participants had an abnormal WHR, particularly those with diabetes. Women, especially those with diabetes, had a higher chance of having an abnormal WHR. Being over 50 and having a higher BMI increases the likelihood of having an abnormal WHR. Diabetic patients often had abnormal levels of HDL and TG. These results highlighted how WHR can help assess metabolic health, especially in people with diabetes.

## Data Availability

The study protocol and the datasets analyzed are available from the corresponding author upon request.
